# The Gender Pay Gap: Income Inequality Over Life Course – A Multilevel Analysis

**DOI:** 10.3389/fsoc.2021.815376

**Published:** 2021-12-23

**Authors:** Lisa Toczek, Hans Bosma, Richard Peter

**Affiliations:** ^1^ Department of Medical Sociology, Institute of the History, Philosophy and Ethics of Medicine, Faculty of Medicine, University of Ulm, Ulm, Germany; ^2^ Department of Social Medicine, Care and Public Health Research Institute (CAPHRI), Faculty of Health, Medicine and Life Sciences, Maastricht University, Maastricht, Netherlands

**Keywords:** employment biographies, gender inequality, income trajectories, LidA-study, growth curve analysis, life course perspective, trajectories of labor market factors

## Abstract

The gender pay gap has been observed for decades, and still exists. Due to a life course perspective, gender differences in income are analyzed over a period of 24 years. Therefore, this study aims to investigate income trajectories and the differences regarding men and women. Moreover, the study examines how human capital determinants, occupational positions and factors that accumulate disadvantages over time contribute to the explanation of the GPG in Germany. Therefore, this study aims to contribute to a better understanding of the GPG over the life course. The data are based on the German cohort study lidA (living at work), which links survey data individually with employment register data. Based on social security data, the income of men and women over time are analyzed using a multilevel analysis. The results show that the GPG exists in Germany over the life course: men have a higher daily average income per year than women. In addition, the income developments of men rise more sharply than those of women over time. Moreover, even after controlling for factors potentially explaining the GPG like education, work experience, occupational status or unemployment episodes the GPG persists. Concluding, further research is required that covers additional factors like individual behavior or information about the labor market structure for a better understanding of the GPG.

## 1 Introduction

In the European Union (EU) in 2019, women’s average gross hourly earnings were 14.1% below the earnings of men (Eurostat, 2021a). The gender pay gap (GPG) has existed for decades and still remains to date. According to Eurostat GPG statistics, the key priorities of gender policies are to reduce the wage differences between men and women at both the EU and national levels (Eurostat, 2021a). Nevertheless, the careers of men and women differ considerably in the labor market, with women being paid less than men (Arulampalam et al., 2005; Radl, 2013; Boll et al., 2017). A report from the European Parliament in 2015 about gender equality assessed Germany’s performance in that field as mediocre. The federal government in Germany has already improved laws that focus on gender equality (Botsch, 2015). Regarding Germany, in 2019 the earning difference between men and women were found to be 19.2% (Eurostat, 2021a). The reasons behind gender income inequality are complex and have multidimensional explanations.

### 1.1 Determinants of the GPG

The early 1990s represented a turning point for the participation of women in the labor market (Botsch, 2015). In previous years, women’s participation rate in the workforce has strongly increased, from 51.9% in the year 1980 (West Germany) to 74.9% in 2019 (OECD, 2021). This upward trend represents the increase of women working at older ages (Sackmann, 2018). However, the gender income inequality remains. Different explaining factors of the GPG were found in previous research: patterns of employment, access to education and interruptions in the careers of men and women.

Although there are nearly equal numbers of men and women in the labor market, when considering women’s careers, various gender-specific barriers are occurring. The working patterns were found to have a relevant impact on the GPG in previous research. Atypical employment is increasing and this result in an expansion of the low-wage sector, which mainly affects women in Germany (Botsch, 2015). Additionally, labor market integration of women has mainly been in jobs that provide few working hours and low wages (Botsch, 2015). Moreover, part-time employment represents a common employment type in Germany, which is more frequent among women – as various studies have demonstrated – and explains the GPG significantly (Boll et al., 2017; Ponthieux and Meurs, 2015; Boll and Leppin, 2015). In addition, the part-time employment occurs more often in occupations characterized by a high proportion of women and low wages (Matteazzi et al., 2018; Boll and Leppin, 2015; Hasselhorn, 2020; Manzoni et al., 2014). Another employment type with few working hours and low pay is a special form of part-time work: marginal work. Marginal work is defined as earnings up to 450 Euros per month or up to 5.400 Euros annually. Also, it is also more common among women than among men (Botsch, 2015; Broughton et al., 2016). The marginal part-time work has increased in nearly all EU countries, especially in Germany where it can be found to be above the EU average (Broughton et al., 2016). Besides the working time, occupational status influences the wage differences of men and women. Female-dominated occupational sectors are characterized by lower wages compared to male-dominated ones (Brynin and Perales, 2016). Additionally, in women-dominant industries, remunerations are less attractive and it often entails low-status work in sectors like retail, caregiving or education (Boll and Leppin, 2015; Hasselhorn, 2020; Matteazzi et al., 2018; Brynin and Perales, 2016). Hence, working patterns such as the amount of working time or the occupational status are crucial determinants that contribute to explaining the GPG in Germany (Blau and Kahn, 2017; Boll et al., 2017).

The access to education and vocational training are important factors, that influence the GPG. Both influence a first access to the labor market and are considered to be ‘door openers’ for the working life (Manzoni et al., 2014). In Germany, education represents a largely stable variable over time, i.e. only few individuals increase their first educational attainment. Education influences the careers of men and women and can be seen as important an determinant of future earnings (Boll et al., 2017; Bovens and Wille, 2017). Although women’s educational attainment caught up with those of men’s in recent years, for men, a higher qualification was still rewarded more than for women (Botsch, 2015; Boll et al., 2017). Moreover, in previous research the impact of education on the GPG was not found to be consistent with different influences for men than for women (Aisenbrey and Bruckner, 2008; Ponthieux and Meurs, 2015). Manzoni et al. (2014) found out, that the effect of education on career developments were dependent of their particular educational levels. In addition, regardless of the women’s educational catching-up in the last years, looking at older cohorts – born between 1950 and 1964 – women had a lower average level of education than men (Boll et al., 2017).

An increasing GPG over time can also be the result of interruptions in careers, which are found more often for women than for men (Eurostat, 2021a; Boll and Leppin, 2015). Previous research of Boll and Leppin (2015) has identified explanations for the GPG in Germany by analyzing data from the German Socio-Economic Panel (SOEP) in 2011. They demonstrated that the amount of time spent in actual work was lower for women than for men. Therefore, women gain less work experience than their male counterparts (Boll and Leppin, 2015). Career interruptions not only impact the accumulation of work experience but also the scope of future work. Especially in the period of family formation higher rates of part-time employment among women can be observed (Boll et al., 2017; Ponthieux and Meurs, 2015). Moreover, work-life interruptions such as raising children or caring for family members have a major impact on the employment development and are more likely to appear for women than for men (Ponthieux and Meurs, 2015). Although the employment rate of mothers has increased in recent years in Germany, it is still considerably lower than that of fathers (Federal Statistical Office, 2021). Hence, taking care of children is still attributed to mothers, to the detriment of their careers (Botsch, 2015). A recent study, however, found sizable wage differences between men and women who were not parents, refuting the assumption that the GPG applies only to parents (Joshi et al., 2020). Other interruptions in the working lives of men and women are caused by unemployment. Azmat et al. (2006) found that in Germany, transition rates from employment to unemployment were higher for women than for men. Career interruptions have lasting negative effects on women’s wages. Therefore, it can be useful to examine unemployment when analyzing gender inequality in the labor market (Eurostat, 2021b).

### 1.2 Theoretical Background

#### 1.2.1 Human Capital Model

In previous research, economic theories had been applied to explain the income differences of men and women. Two essential factors could be found: qualification and discrimination. The human capital model claims that qualifications with greater investments can be directly related to higher wages of men and women. The earnings are assumed to be based on skills and abilities that are required through education and vocational training, and work experience (Grybaitė, 2006; Lips, 2013; Blau and Kahn, 2007). Educational attainment of women has caught up in recent years (Botsch, 2015). However, women’s investments in qualifications were still not equally rewarded as those of men. Therefore, the expected narrowing of the GPG was not confirmed in earlier research (Boll et al., 2017; Lips, 2013). Another determinant of the human capital model is work experience. Labor market experience contributes to a large extent to the gender inequality in earnings (Sierminska et al., 2010). Hence, work experience influences the wages of men and women. On the one hand, interruptions due to family life lower especially women’s labor market experience compared to men. On the other hand, part-time employment is more frequent among women with fewer working hours and therefore less work experience. The lesser accumulation of work experience leads to lower human capital and lower earnings for women compared with men (Blau and Kahn, 2007; Mincer and Polachek, 1974). Nonetheless, the association of work experience and income is more complex. Regarding the wages of men and women the influence of occupation itself also needs to be considered (Lips, 2013). In the paper of Polachek (1981) different occupations over the careers of men and women were explained by different labor force participation over lifetime. Referring to the human capital model, it is argued that women more likely expect discontinuous employment. Therefore, women choose occupations with fewer penalties for interruptions (Polachek, 1981). However, it should be questioned if working in specific occupations can be defined as a simple choice (Lips, 2013). Besides, part-time employment is found to be more frequent among women, which ultimately leads to few working hours and hence low earnings (Botsch, 2015; Ponthieux and Meurs, 2015; Boll et al., 2017). Though different working hours cannot be defined as a simple choice either (Lips, 2013).

Earlier criticism about the human capital model discussed that the wage differences of men and women cannot only be explained by the qualification and the labor market experience (Grybaitė, 2006; Lips, 2013). Another theoretical approach explaining the GPG refers to labor market discriminations, which effect occupations and wages (Boll et al., 2017; Grybaitė, 2006). On the one hand, occupational sex segregation can be associated with income differences of men and women. The different occupational allocation in the labor market of men and women are defined as allocative discrimination (Petersen and Morgan, 1995). In addition, occupations in female-dominated sectors are mostly characterized by low-wages compared to more male-dominated occupations (Brynin and Perales, 2016). On the other hand, even with equal occupational positions and skill requirements women mostly earn less than men, this refers to the valuative discrimination (Petersen and Morgan, 1995). Even within female-dominated jobs a certain discrimination exists, with men being paid more than women for the same occupation. Additionally, employment sectors with a large number of female workers are more likely to be associated with less prestige and lower earnings (Lips, 2013). Achatz et al. (2005) analyzed the GPG with an employer-employee database in Germany. The authors examined the discrimination in the allocation of jobs, differences in productivity-, and firm-related characteristics. They found out that in occupational groups within companies, the wages decreased with a higher share of women in a group. Additionally, a higher proportion of women in a groups resulted in a higher wage loss for women than for men (Achatz et al., 2005).

Although relevant criticism of the human capital model exists, its determinants are still found to be important in explaining the wage differences of men and women (Boll et al., 2017). Nonetheless, income differences of men and women can still be found even with the same investments in human capital. The reason for this could be the occupational discrimination of women (Brynin and Perales, 2016; Achatz et al., 2005; Lips, 2013). Therefore, the occupational positions can be associated as a relevant factor of the GPG.

#### 1.2.2 Life Course Approach

Besides economic theories, there are other theoretical approaches of explaining the GPG. One of them focusses on the accumulation of disadvantages over the life course: the ‘cumulative advantage/disadvantage theory’ by Dannefer (2003). It also involves social inequalities which can expand over time. The employment histories of men and women evolve over their working lives and during different career stages, advantages and disadvantages can accumulate. First, this life course perspective considers and underlines the dynamic approach of how factors shape each individual life course. Secondly, it can contribute to explain the different income trajectories of men and women over their working lives (Doren and Lin, 2019; Dannefer, 2003; Härkönen et al., 2016; Manzoni et al., 2014; Barone and Schizzerotto, 2011).

The importance of the life course perspective was underlined by some earlier studies. They demonstrated that certain conditions in adolescence or early work-life affected future careers of men and women. Visser et al. (2016) found evidence for an accumulation of disadvantages in the labor market over working life, in particular for the lower educated. The cohort study SHARE had assessed economic and social changes over the life course in numerous European countries in several publications (Börsch-Supan et al., 2013). Overall, education and vocational training, occupational positions and income illustrate parts of the social structure which in turn can demonstrate gender inequality in the labor market (Boll and Leppin, 2015; Hasselhorn, 2020; Du Prel et al., 2019). Moreover, family events and labor market processes repeatedly affect one another over the life course. The work-family trajectories have consequences on employment outcomes such as earnings (Aisenbrey and Fasang, 2017; Jalovaara and Fasang, 2019). Furthermore, the income differences of men and women are not steady but tend to be lower at the beginning of employment and increase with age (Goldin, 2014; Eurostat, 2021a). Therefore, careers should not be analyzed in a single snapshot, but with a more appropriate life course approach that takes into account factors that influences the wages of men and women over time.

### 1.3 Aim and Hypotheses

The aim of the present study is to examine income trajectories and to investigate the income differences of men and women over their life course. We are interested in how human capital determinants, occupational positions and the accumulation of disadvantages over time contribute to the explanation of the GPG from a life course perspective.

Focusing on older German employees, our study includes 24 years of their careers and considers possible cumulative disadvantages of women in the labor market compared to those of men. In contrast to Polachek (1981), who analyzed the GPG as a unit over lifetime, we used a life course approach in regard to the theory of cumulative disadvantages of Dannefer (2003). Accordingly, we analyze explaining factors of the GPG not only in a single snapshot but over the working careers of men and women. Life course data based on register data and characteristics of employment biographies with information on a daily basis are two additional important and valuable advantages of our study. Existing studies rarely have this information in the form of life course data and when they do, the data is either self-reported and retrospective including possible recall bias, or based on register data which was only collected on a yearly basis. We expect to find differences in the income of men and women over a period of time with overall higher, and more increasing earnings of men than of women.

Hypothesis 1 (H1): The differences of income trajectories throughout working life is expected to demonstrate more income over time among men than among women.

Education and vocational training, and work experience are human capital determinants. They have influence on the earnings of men and women. Although previous research estimated additional important factors contributing to the GPG, human capital capabilities continue to be relevant in explaining the wage differences of men and women (Blau and Kahn, 2007; Boll et al., 2017). In our life course approach, we control for human capital determinants due to the information about education and vocational training, and work experience via the amount of working time (full-/part-time) for each year. We expect to find a strong influence of both determinants on the wages of men and women in Germany.

Hypothesis 2 (H2): The income differences between men and women can be explained by determinants of the human capital model.

Previous research found out that factors such as occupational status had an impact on the income differences of men and women (Blau and Kahn, 2007; Boll et al., 2017). For a better understanding and explanation of the GPG, gender differences regarding occupational positions must be included to human capital determinants (Boll et al., 2017). We assume that men and women can be found in different occupations, measured via occupational status, and these explain a substantial part of the wage differences between men and women.

Hypothesis 3 (H3): The occupational status of men and women can contribute to the explanation of the GPG.

The life-course approach acknowledges time as an important influence on the wages of men and women. Income differences of men and women can change over time and career stages, while the GPG was found to be lower at the beginning of the employment career and widened with age (Goldin, 2014). Hence, the earning differences between men and women tend to be higher for older employees (Eurostat, 2021a; Federal Statistical Office, 2016). To account for the influence of age, we additionally included the age of each person in our analysis. Another factor that changes over time and contribute to explain the GPG is part-time work. In general, part-time work result in a disadvantage in pay compared to full-time employment (Ponthieux and Meurs, 2015). However, explanations of the GPG due to different amount of part-time work need to include a special form of part-time work: marginal work. Marginal employment conditions are characterized by low wages and high job insecurities. Also discontinuous employment due to unemployment are characterized by job insecurities and affect the low-paid sector – therefore mainly women (Botsch, 2015). Besides the human capital determinants and occupational positions as important factors explaining the GPG, the region of employment influences the wages of men and women and can also change over the career stages. Evidence from the Federal Statistical Office of Germany in 2014 noticed a divergence of the GPG trend in the formerly separated parts of Germany. The GPG among employees was wider in the Western part (24%) compared to the Eastern part of Germany, where it was found to be 9% (Federal Statistical Office, 2016). Therefore, to examine income differences, the amount of less advantaged employment such as marginal work or periods of unemployment throughout the careers of men and women needs to be considered, as well as the region of employment and the age of a person.

Hypothesis 4 (H4): Factors of the living environment such as regional factors, and social disadvantage work conditions such as marginal work or unemployment, contribute to the income difference between men and women.

Our study about the GPG in Germany adds to earlier research in different ways. First, the accumulation of inequalities over the life course of men and women is known, but only few studies exist that focus on income through life course approach. We can analyze factors that influence the GPG over the careers of men and women due to the availability of social security data with daily information of each person. Besides the wages of men and women, the data additionally contains time-varying information about occupational status, working time and unemployment breaks. Therefore, we use longitudinal data of the German baby-boomers which allow us to measure changes of factors explaining the GPG over time. Second, a relevant contribution of our study is that we can consider different factors contributing to the explanation of the GPG through a life course perspective. The few studies focusing on the GPG over life course included either only determinants of the human capital model (Joshi et al., 2020) or factors of occupational careers (Moore, 2018). Some research included both aspects but had other disadvantages, such as Monti et al. (2020), who could not analyze temporal evolution of the GPG with the data available. Moreover, previous research on the GPG in Germany could not trace vertical occupational segregation due to missing information of part-time workers, included only data of West Germany and used merely accumulated earnings over time (Boll et al., 2017). Nonetheless, previous research demonstrated the need of analyzing the GPG via life course approach with which the accumulation of advantages and disadvantages for both, men and women, can be considered. Third, due to the usage of a multilevel framework we can examine income trajectories simultaneously at an individual and at a time-related level. Moreover, the influences of time-invariant and time-varying factors can be analyzed regarding differences in earnings of men and women. Hence, the multilevel approach examines income changes between and also within individuals. Furthermore, it acknowledges the importance of the life course perspective with including time as a factor in the model. A recent study also used growth curve modelling to explain gender inequality in the US. However, gender inequality measured through gender earnings was analyzed only across education and race without considering other variables explaining the GPG (Doren and Lin, 2019). To our knowledge, there exists no research on the GPG that covers several essential determinants, hence we aim to fill those research gaps with our study.

## 2 Materials and Methods

### 2.1 Sample

The data were obtained from the cohort study lidA (living at work). The lidA sample includes two cohorts of employees (born in 1959 and in 1965) and was drawn randomly from social security data. LidA combines two major sources of information – register data of social insurance and questionnaire data derived from a survey. The survey was conducted in two waves, 2011 (t_0_) and 2014 (t_1_) (Hasselhorn et al., 2014). The ethics commission of the University of Wuppertal approved the study.

**FIGURE 1:**
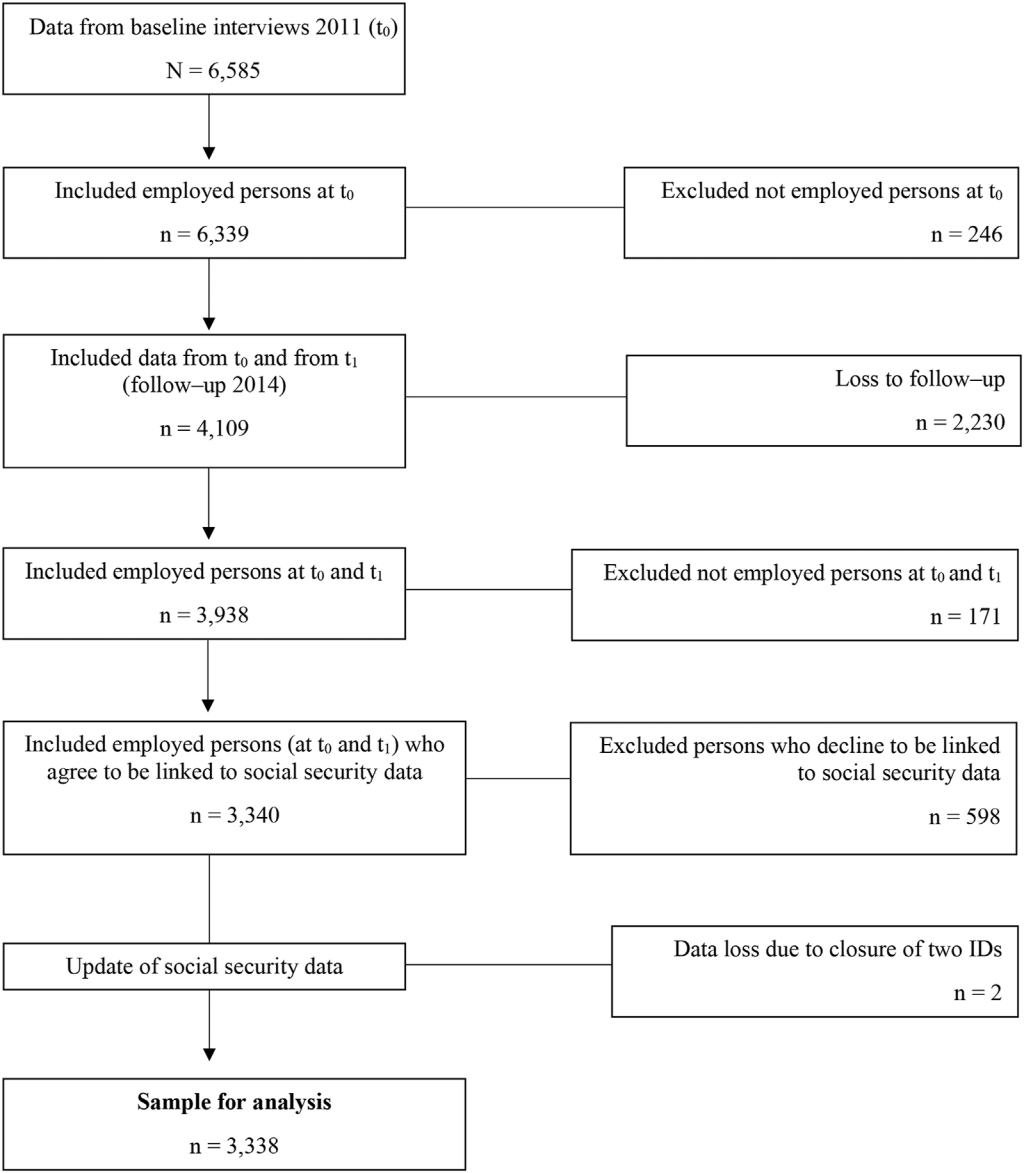
Decision tree – inclusion and exclusion criteria in the sample for analysis.

In Germany, the social insurance system assists people in case of an emergency such as unemployment, illness, retirement, or nursing care. Employees have to make a contribution to the system depending on their income – except of civil servants or self-employed (Federal Agency for Civic Education, 2021). In our analyses, we included men and women in Germany who participated in the baseline (2011) and in the follow-up (2014), were employed during both waves and subjected to social security contributions. We only included persons who agreed via written consent to the linkage of the survey data to their social security data. Thus, our sample for analysis included 3,338 individuals (Figure 1).

### 2.2 Measurements

The social security data of the Institute for Employment Research of the German Federal Employment Agency is based on employers’ reports. The so-called “Integrated Employment Biographies” (IEB) or register data comprises information about individual employment; that is, type of employment, occupational status, episodes of unemployment and income with information about age, gender and education and vocational training. The IEB data are retrieved from employers’ yearly reports submitted to the social security authority (Hasselhorn et al., 2014). The information of the register data was available on a daily basis and contained yearly information from 1993 to 2017 for each person. However, the IEB data contain missing details, especially regarding information that is not directly relevant for social security data and therefore, not of the highest priority for employers’ reports. This is particularly true for data on gender and education and vocational training. As our sample participants consented to the linkage of IEB with questionnaire data, we were able to impute the missing information on these variables with the help of the survey data. All time-varying information in the IEB is coded to the day. Our data have a multilevel structure with time of measurements (Level 1) being nested within individuals (Level 2) and defined as follows.

#### 2.2.1 Level 1 Variables

In our analysis the variable time was based on information about the year of measurement. The starting point represents 1993 and was coded with zero. The outcome variable income was calculated from the IEB data as nominal wages in Euros (€). As time-varying variable, it can be defined as the average daily income per year of each person whose work contributes to social security and/or marginal employment. Information about the work experience due to working time was available for jobs that require social security contribution. To draw this information from the IEB data, the time-varying variable working time was computed with three different types: full- and part-time, part-time, and full-time. The data on occupational status were based on the International Standard of Classification of Occupations 2008 (ISCO-08). This time-varying variable contained information on the occupational status of each job that a person has held over the years. For the multilevel analysis, ISCO-08 was transformed from the German classification KldB 2010 (classification of occupations 2010) of the register data. ISCO-08 is structured according to the skill level and specialization of jobs, which are grouped into four hierarchical levels. Occupational status in our study was defined by the 10 major groups (level one of the classifications ISCO-08), without the group of armed forces who did not appear in our data. Therefore, the nine groups were analyzed: elementary occupations; plant and machine operators and assemblers; craft and related trades workers; skilled agricultural, forestry and fishery workers; services and sales workers; clerical support workers; technicians and associate professionals; professionals; and managers (International Labour Office, 2012). Moreover, information about the number of episodes of marginal work could also be drawn from the register data. Marginal work was defined due to having at least one marginal employment per year. The time periods (episodes) of every marginal employment were counted and added up yearly. Furthermore, the duration of unemployment as time-varying variable was calculated due to information of the register data about the days of unemployment per year. In the register data unemployment is defined as being unemployed or unable to work for up to 42 days, excluding those with sickness absence benefits or disability pensions. The IEB data also provided information on the region of employment, which represents the area in which a company is located (East Germany and West Germany). This time-varying variable was available for each person over the years. A description of the Level 1 characteristics of our sample is provided in Table 2 using the last available information (2017) from the IEB data.

#### 2.2.2 Level 2 Variables

Information about the time-invariant variable education and vocational training was assessed from the survey data in 2011 (baseline). Education and vocational achievements of the sample were grouped in: low, intermediate and high education and vocational training (see Supplementary Table S1). The time-invariant variable gender had missing values in the register data. Therefore, we imputed the missing data using information of the survey data. The variable was coded 0 = female and 1 = male. Also based on the survey data, we included the time-invariant variable year of birth with measurements of 1959 and 1965 in the analysis. The characteristics of the Level 2 variables are displayed in Table 1.

**TABLE 1:** Characteristics of the Level 2 variables^a^ for men (n = 1,552) and women (n = 1,786).

Variables	Men	Women	—
	n (%)	n (%)	Cramer’s V
Education and vocational training	—		0.15***
Low	405 (26.1)	307 (17.2)	—
Intermediate	750 (48.3)	1,124 (62.9)	—
High	395 (25.5)	354 (19.8)	—
Missing	2 (0.1)	1 (0.1)	—
Year of birth	—	—	0.02
1959	678 (43.7)	815 (45.6)	—
1965	874 (56.3)	971 (54.4)	—

a The database of the variable is provided by survey data in 2011.

*p < 0.05, **p < 0.01, ***p < 0.001.

### 2.3 Statistical Analysis

The characteristics of our sample are displayed in Table 1 and Table 2. Statistical analyses were performed using either Cramer’s V or by unpaired two sample *t*-test for numeric variables. Regarding the multilevel analysis, we used a so-called growth curve analysis. It demonstrates a multilevel approach for longitudinal data that model growth or decline over time. For this purpose, all daily information in the IEB were transformed into data on a yearly basis. Level 1 (year of measurements) represents the intraindividual change with time-varying variables. Interindividual changes are determined with time-invariant variables on Level 2 (individuals). Therefore, time of measurements predictors was nested within individuals. We applied a random intercept and slope model, which assumed variations in intercept and slope of individuals over time (Singer and Willett, 2003; Rabe-Hesketh and Skrondal, 2012; Hosoya et al., 2014). Besides the Level 1 and Level 2 predictors, the cross-level interaction of gender*time interaction was constituted to analyze differences in income slopes of men and women over time (Rabe-Hesketh and Skrondal, 2012).

**TABLE 2:** Characteristics of Level 1 variables^a^ for men (n = 1,552) and women (n = 1,786).

	Men	Women	—
Variables	n (%) or M±SD (n)	n (%) or M±SD (n)	Cramer’s V or t-value
Occupational status (ISCO)	—	—	0.40***
Elementary occupations	48 (3.1)	53 (3.0)	—
Plant and machine operators and assemblers	200 (12.9)	66 (3.7)	—
Craft and related trades workers	313 (20.2)	51 (2.9)	—
Skilled agricultural, forestry and fishery workers	18 (1.2)	5 (0.3)	—
Services and sales workers	83 (5.3)	229 (12.8)	—
Clerical support workers	135 (8.7)	324 (18.1)	—
Technicians and associate professionals	286 (18.4)	463 (25.9)	—
Professionals	248 (16.0)	327 (18.3)	—
Manager	95 (6.1)	55 (3.1)	—
Missing	126 (8.1)	213 (11.9)	—
Average daily income per year	—	—	26.23***
Average daily income	137.94 ± 52.1 (1447)	90.00 ± 49.4 (1668)	
Missing	105 (6.8)	118 (6.6)	
Working time	—	—	0.54***
Full- and part-time	6 (0.4)	33 (1.8)	—
Part-time	83 (5.3)	865 (48.4)	—
Full-time	1,337 (86.1)	675 (37.8)	—
Missing	126 (8.1)	213 (11.9)	—
Region of employment	—	—	0.02
Eastern Germany	261 (16.8)	327 (18.3)	—
Western Germany	1,186 (76.4)	1,341 (75.1)	—
Missing	105 (6.8)	118 (6.6)	—
Numbers of episodes of marginal work	0.09 ± 0.3 (1,552)	0.18 ± 0.5 (1,786)	−6.54***
Duration of unemployment	6.35 ± 43.6 (1,552)	7.32 ± 46.0 (1,786)	−0.62

*M* mean; *SD* standard deviation.

a The database of the variable is provided by IEB, data in 2017.

*p < 0.05, **p < 0.01, ***p < 0.001.

Level 1 of the two-level growth model is presented below (Eq. (1)). $y_{ij}$ measures the income trajectory y for individual i at time j . True initial income for each person is represented with $\beta_{0i}$ . The slope of the individual change trajectory demonstrates $\beta_{ij}$ . $TIME_{ij}$ stands for the measure of assessment at time j for individual i (Level 1 predictor). The residual or random error, specific to time and the individual is demonstrated by $\varepsilon_{ij}$ .

$$y_{ij}=\beta_{0i}+\beta_{1i}TIME_{ij}+\varepsilon_{ij}$$ (1)

Eq. 2 and 3 represent the submodels of the Level 2. Eq. 2 defines the intercept $\gamma_{00}$ for individual i with the intercept of $z_{i}$ (illustrating a Level 2 predictor) and residual in the intercept $v_{0i}$ . The slope at Level 2 is represented in Eq. 3 with $\gamma_{10}$ and the slope error $v_{1i}$ . The effect $\gamma_{11}$ provides information on the extent to which the effect of the Level 1 predictor ( $TIME_{ij}$ ) varies depending on the Level 2 predictor ( $z_{i}$ ).

$$\beta_{0i}=\gamma_{00}+\gamma_{01}z_{i}+v_{0i}$$ (2)

$$\beta_{1i}=\gamma_{10}+\gamma_{11}z_{i}+v_{1i}$$ (3)

To test our hypotheses, we calculated the influence of different variables with adjusting various predictors stepwise into the multilevel analysis. First, we estimated an unconditional means model which describes the outcome variation only and not its change over time (model 1). The next preliminary step was calculating the intraclass correlation coefficient (ICC) of this model 1. It identifies and partitions the two components: within- and between-person variance. The ICC estimates the proportion of total variation of the outcome y that lies between persons (Singer and Willett, 2003). In the next model (model 2), we calculated an unconditional growth curve model which included time as predictor on Level 1. In model 3, the GCA was controlled for gender and time as well as the interaction of both variables. Model 4 was additionally adjusted for human capital determinant: education and vocational training, and working time. The GCA of model 5 was controlled for occupational status. The last model included year of birth, number of episodes of marginal work, duration of unemployment and region of employment (model 6 – fully adjusted model).

In Table 5, the indices of the Akaike’s Information Criterion (AIC) were used to compare models and explore the best model fit (Singer and Willett, 2003; Rabe-Hesketh and Skrondal, 2012). The statistical analyses were performed with IBM SPSS 25.

## 3 Results

### 3.1 Descriptive

Characteristics of Level 2 variables stratified by gender are displayed in Table 1. 1,552 men and 1,786 women were included in the analyses. It is observed that women significantly differ from men in education and vocational training. Women were less likely than men to have both low and high levels of education and vocational training.

The characteristics of Level 1 variable are represented in Table 2. Men and women differ significantly in their occupational positions. Also, men had a higher average daily income than women. Part-time jobs are more likely among women as compared to men, who are more likely to be represented in full-time jobs. Moreover, the numbers of episodes of marginal work differ significantly between men and women.

**FIGURE 2:**
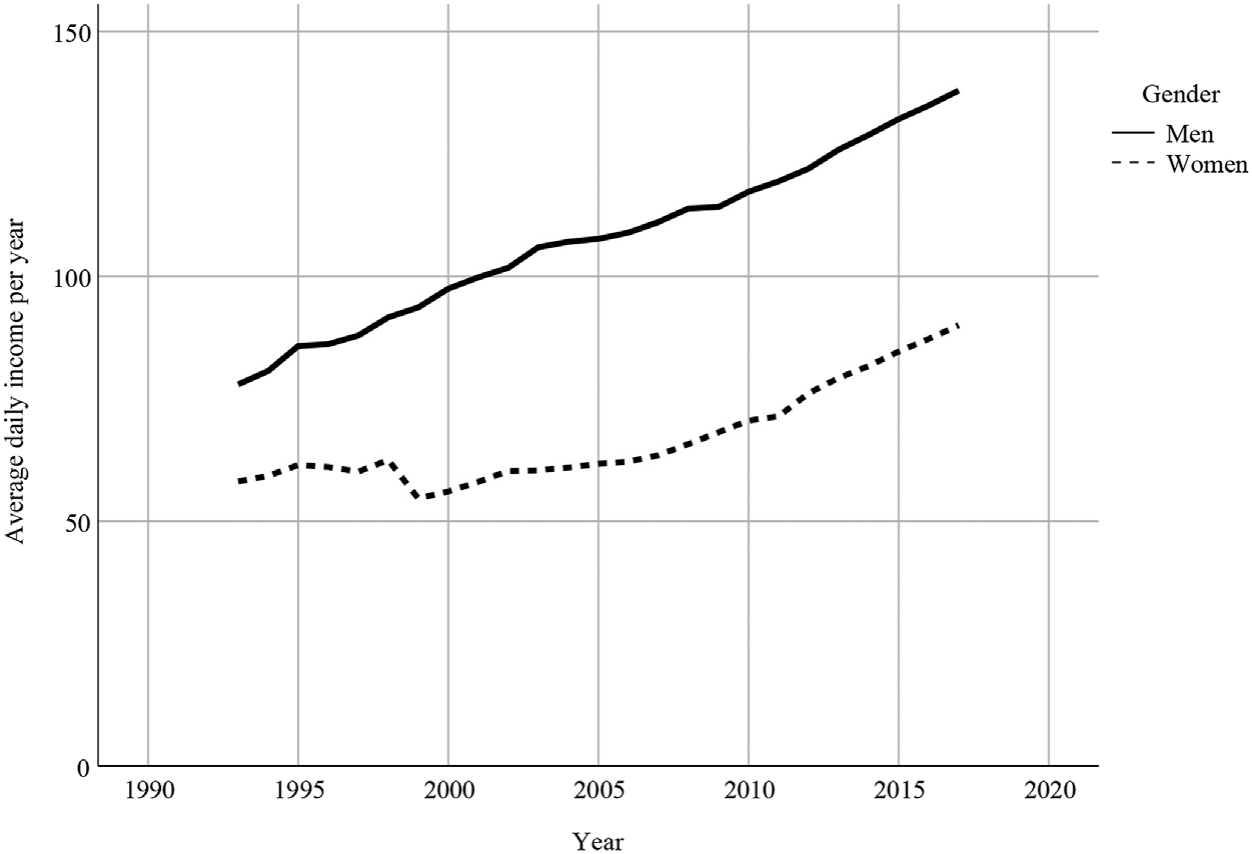
Income trajectories of men and women.

Figure 2 displays the income trajectories over the observation period (1993–2017) among men and women. In 24 years, average daily income per year increased for both. However, men have a higher average income over their life course than women. Over time, a steeper growth of the average daily income per year can be observed for men, compared to the income development of women.

### 3.2 Growth Curve Analysis

Results of the multilevel analyses with average daily income per year as dependent variable concerning H1 are presented in Table 3. The ICC of the unconditional means model (model 1) demonstrates that 74% of the total variability in income can be attributed to differences between persons and 26% to the differences within persons. Adding time as a predictor in the multilevel analysis (model 2), the variance components on Level 1 become smaller. Concluding that time accounts for 68% (from 607.34 to 197.12) of the within-person variance in average income. On Level 2, time explains 40% of the variance between persons (interindividual). However, there can be still found significant unexplained results in both levels which suggests that predictors on both levels should be further included. The GCA in model 3 was adjusted for gender (with women as reference group) and the interaction gender*time. The results show a significant effect of gender on the average income over time. The starting place (intercept) lies at 41.74€ with an incremental growth per year of 1.76€. However, regarding women as reference group, men have a higher average income. The significant interaction term also indicates different income development of men and women over time – with men having higher average income trajectory than women. As expected, no relevant change can be found in the within-person variance due to the adding of the Level 2 variable: gender. The variance on Level 2, however, become less concluding that gender accounts for 26% of the variance between persons. Overall, we can verify H1 with these results.

**TABLE 3:** Growth curve models 1 to 3: Estimates of average daily income per year.

	Model 1^a^	Model 2^b^	Model 3^c^
Fixed effects	Coefficient (S.E.)		
Intercept	84.70*** (0.72)	58.23*** (0.61)	41.74*** (0.73)
Time (year of measurement)	—	2.06 *** (0.04)	1.76*** (0.05)
Gender (ref.: women)	—	—	35.50*** (1.06)
Gender by time	—	—	0.66*** (0.07)
Variance components			
Within-person (L1)	607.34*** (3.22)	197.12*** (1.07)	197.15*** (1.07)
In intercept (L2)	1,682.35*** (41.92)	1,202.55*** (31.49)	884.60*** (23.57)
In rate of change (L2)	—	4.10*** (0.11)	3.99*** (0.10)

L1 = Level 1; L2 = Level 2.

a Unconditional Means Model.

b Unconditional Growth Model.

c Model controlled for gender, time, and interaction gender by time.

Results of the GCA with average daily income per year as the dependent variable controlled by determinants of the human capital model are presented in Table 4 (model 4). In addition to the multilevel analysis of model 3, model 4 is also adjusted for: education and vocational training, and working time. The results show that the average income is found to be significantly higher for full-time workers and higher educated. There is a social gradient for income regarding education and vocational training – with decreasing levels of education, the income also reduces. People who are working full-time have a higher average income than those who work part-time or full- and part-time. The effect of gender is found to be significant with less average income of women compared to men. Moreover, the income development of men and women over time is still significantly different, with more income growth over time for men than for women. The results of the variance components demonstrate that human capital determinants are explaining 16% of the variance within person and 25% of the variance between persons. However, on both levels there can be still found significant variance and additional variables need to be considered. Our hypothesis 2 can be partially confirmed.

**TABLE 4:** Growth curve models 4 to 6: Estimates of average daily income per year.

	Model 4^a^	Model 5^b^	Model 6^c^
Fixed effects	Coefficient (S.E.)		
Intercept	64.85*** (1.10)	64.88*** (1.28)	48.57*** (1.63)
Time (year of measurement)	1.92*** (0.05)	1.90*** (0.05)	1.90*** (0.04)
Gender (ref.: women)	26.16*** (0.95)	26.37*** (0.94)	25.86*** (0.90)
Gender by time	0.56*** (0.07)	0.57*** (0.07)	0.58*** (0.06)
Education			
High (ref.)	0	0	0
Intermediate	−14.67*** (1.13)	−13.74*** (1.13)	−13.67*** (1.07)
Low	−21.58*** (1.37)	−19.76*** (1.40)	−21.59*** (1.30)
Working time			
Full-time (ref.)	0	0	0
Part-time	−16.10*** (0.25)	−16.19*** (0.25)	−16.31*** (0.25)
Full- and part-time	−6.43*** (0.44)	−6.41*** (0.44)	−5.55*** (0.44)
Occupational status			
Manager (ref.)	—	0	0
Professionals	—	1.16 (0.71)	1.22 (0.70)
Technicians and associate professionals	—	1.57* (0.70)	1,50* (0.69)
Clerical support workers	—	−2.15** (0.71)	−2,05** (0.70)
Services and sales workers	—	−1.95** (0.75)	−2,07** (0.74)
Skilled agricultural, forestry and fishery workers	—	−5.25*** (1.35)	−4,52*** (1.33)
Craft and related trades workers	—	−2.18** (0.77)	−2,34** (0.76)
Plant and machine operators and assemblers	—	−2.32** (0.80)	−2,32** (0.79)
Elementary occupations	—	−2.43** (0.93)	−2,26* (0.92)
Year of birth (ref.: 1965)	—	—	5.21*** (0.85)
Number of episodes of marginal work	—	—	−5.21*** (0.22)
Duration of unemployment	—	—	−0.05*** (0.00)
Region of employment (ref. East)	—	—	8.51*** (0.57)
Variance components			
Within-person (L1)	166.58*** (0.93)	165.90*** (0.93)	161.911*** (0.91)
In intercept (L2)	662.38*** (18.39)	641.60*** (17.93)	576.81*** (16.24)
In rate of change (L2)	3.25*** (0.09)	3.22*** (0.09)	3.14*** (0.08)

L1 = Level 1; L2 = Level 2.

a Model additionally adjusted for education and vocational training, and working time.

b Model additionally adjusted for occupational status.

c Model additionally adjusted for year of birth, marginal work, duration of unemployment and region of employment.

Model 5 (Table 4) embeds occupational status to the analysis to find out the contribution of the occupational positions on the earning differences of men and women. Significant differences in the daily average income for each occupational group can be identified. The reference group is represented with the highest occupational group ‘manager’. In nearly all other occupations, manager had the highest average income, except of ‘technicians and associate professionals’. Moreover, the effects of occupational status on income are significant for all ISCO groups except for professionals. However, compared to education and vocational training, occupational status trends are less clear, and a social gradient cannot be identified. The estimated of the fixed effect of gender persists and stays the same, concluding that the occupational position of a person could not influence the effect of gender on income. The increase of income over time can be still found to be significant higher for men than for women. Moreover, including the Level 1 variable, occupational position cannot explain a substantial part of the within-person variance. We can identify occupational positions as significant predictor of the income, but a relevant contribution to explain the GPG cannot be observed. Therefore, we cannot approve hypothesis 3.

The results of investigating the influence of factors of the living environment are presented in Table 4 (model 6). Those, who are born earlier (1959) are found to have a higher average daily income, compared to those born in 1965. Having at least one marginal employment per year influences the average daily income negatively, as does having more unemployed days. Furthermore, average income is influenced by the region of employment, being lower in East Germany than in West Germany. The estimate of gender become a little less, but the average income and the development of income over time still substantially differs between men and women. The factors of living environment account for 10% of the variance between persons. We can only partially accept hypothesis 4.

### 3.3 Goodness of Fit

Table 5 displays the goodness of fit statistics for the different models of the GCA. The AIC is computed to find the best model fit. Considering the different indices of AIC, model 6 has the best fit.

**TABLE 5:** Goodness-of-fit statistics of the GCA.

	Model 1^a^	Model 2^b^	Model 3^c^	Model 4^d^	Model 5^e^	Model 6^f^
AIC	702,153.84	631,357.37	630,223.72	585,341.46	583,256.61	581,243.22

AIC Akaike’s Information Criterion.

a Unconditional means model. Not displayed in detail.

b Unconditional growth model. Not displayed in detail.

c Model controlled for gender, year and the interaction gender*year. See Table 3.

d Model additionally adjusted for education and vocational training, and working time. See Table 4.

e Model additionally adjusted for occupational status. See Table 4.

f Model additionally adjusted for year of birth, number of episodes of marginal work, duration of unemployment (days per year) and region of employment. See Table 4.

## 4 Discussion

This study aimed to examine the income differences of men and women over their life course. We investigated how different factors can explain the GPG over time. Even after extensive control for human capital determinants, occupational factors and various factors of the living environment, the effect of gender on the average daily income persisted. Moreover, the average income development was found to be higher for men compared to women.

The accumulation of inequalities over time can be seen in the difference between men’s and women’s wages. Over the period of 24 years, our results showed that the income development of men increased more compared to women – the GPG widened with time. Due to the availability of life course data, we could consider cumulative disadvantages regarding the earnings of men and women. Moreover, the results of the variance componence also showed the importance of including time to explain the GPG (Table 3, model 2). Therefore, we can verify our first hypothesis. The steeper incline of income for men compared to women over time substantiates the presence of GPG in Germany. Goldin (2014) also found a small GPG when people enter the labor market and a widening gap with age. Our findings are also in line with information from the Federal Statistical Office (2016) and Eurostat (2021a) who used representative data and not use cohort specific data of the German working population.

The second hypothesis assumed that human capital determinants (education and work experience) can explain the GPG. The effects of education and vocational training on daily average income significantly differed in our results (Table 4, model 4). Findings of Bovens and Wille (2017) also demonstrated that the level of a person’s education determines the income level. Our results also support the previous finding, that education is most often a requirement for the achievement of a certain desired financial situation (Du Prel et al., 2019). Our results also showed that the average income significantly differed considering working time. Full-time workers had higher average income, while men were more likely to work full-time compared to women. Earlier research also showed that part-time work was more frequent among women than among men (Boll and Leppin, 2015; Matteazzi et al., 2018; Eurostat, 2021a). After adjusting for human capital determinants, the unexplained variance was still substantial and the effect of gender remained significant. Hence, H2 can only partially be accepted.

In our third hypothesis, we assumed that the gender differences in occupational position can explain the GPG. We demonstrated that the average income differed according to the occupational status of a person. This is in line with previous findings of Blau and Kahn (2001) who assumed occupation to be an important factor of the financial status of a person. After controlling for occupational status, the effect of gender could still be found to be significant. We cannot accept H3 and therefore cannot confirm results of earlier studies (Blau and Kahn, 2007; Boll et al., 2017). In contrast to the results of education and vocational training, we did not observe a clear social gradient of occupational status and income in our analyses. One explanation could be the classification of the occupational status. The ISCO classification is structured hierarchically on four levels. The construction is based on skill level and specialization. In our study, we used the major group structure (level one) with 10 different occupational groups. Using ISCO at level one (major groups) cannot be interpreted as a strict hierarchical order of occupations; instead, it can be considered more of a summary information on occupational status regarding skill level. Moreover, we were only able to generate the major groups of the register data and therefore cannot provide more detailed information about the occupational status. However, ISCO is applied in our study for the purpose of international comparability (International Labour Office, 2012).

The accumulation of disadvantages over time could also be found in our results after controlling for factors such as unemployment or marginal employment. Having (at least one) marginal employment per year influenced the income negatively. We found that discontinuities in employment and interruptions such as unemployment also had a significant negative effect. Average income decreased when the number of days per year of unemployment increased. Furthermore, controlling for the region of employment, people in East Germany had lower daily average income compared to those in West Germany. Regarding the difference between men and women, previous findings also suggested a wider GPG in West Germany than in East Germany (Federal Statistical Office, 2016). However, the GPG in West and East Germany should be compared with caution due to different societal models in the past. Moreover, different labour market characteristics and different infrastructure of childcare facilities lead to a lower GPG in East Germany than in West Germany (Federal Ministry for Family Affairs, Senior Citizens, Women and Youth, 2020). The year of birth was included to eliminate cohort effects, and it was found to influence average income. Men and women born earlier (1959) had higher income than those born in 1965. The fact that they are older and have worked longer in the labor market could be an explanation. The significant effects of gender on the average income and the income trajectories remained after adjusting for these factors. Therefore, hypothesis 4 can only be partially confirmed.

### 4.1 Strengths and Limitations

Our study has limitations concerning the generalizability of our results due to the database. Our sample includes employees of two age groups (1959 and 1965) in Germany, who are subjected to social security. Thus, the generalizability or extension of the findings to self-employed people, civil servants and other age groups may be limited. The GPG differs considerably between the EU members. The GPG in Germany is one of the widest in the EU, with 19.2% in 2019. Netherlands and Sweden are two EU countries with similar employment rates, but still have lower GPGs with 14.6 and 11.8% (Eurostat, 2021a). Efforts to promote gender equality in politics in Germany are limited compared to other EU members. Women are still underrepresented, not only in the political but also in the economic area. Moreover family policy needs to further support full-time employment of women and working mothers (Andersson et al., 2014; Botsch, 2015). Therefore, the transfer of our results to other countries should be made with caution. There are some other limitations regarding the IEB data. Information about occupational careers exist from the beginning (1975), but only for persons born in West Germany. Information about people born in East Germany was not available for the period before 1993. Hence, to counteract the systematic bias, we defined 1993 as a cut-off point, when people were either 28 or 34 years old. Additionally, we adjusted our analyses for the region of employment (East/West Germany). Furthermore, information about the marginal work and duration of unemployment were only available from 1999 onwards. Due to the composition of the IEB data, we could not include people who were unwell for long periods of time. Only persons who were unable to work for less than 42 days were included in the data. Regarding the income development of women in our study, Figure 2 shows a decrease between 1997 and 1999. Being in their thirties (32–40 years) and having to raise children at that time can be one possible explanation. Regarding family formation, in 1993 the average age of a mother at birth was 28.4 years (Federal Statistical Office, 2020). At the beginning of our analysis (1993) the average age of both cohorts in the study (28 years; 34 years) is similar to the average age of a mother during that time – especially for the younger cohort. However, our data do not cover information about persons on parental leave or homemakers. Due to the lack of information in the IEB data, implications of family life contributing to a difference in pay for women cannot be included in our analysis. Furthermore, Joshi et al. (2020) could not find a GPG only for parents but also for men and women without children. Therefore, the issue of wage differences between men and women is relevant either way.

Besides these restrictions, our study exhibits several strengths. The study population is highly representative for German employees subject to social insurance contributions, born in 1959 and 1965 and is, therefore, characterized by a high external validity (Schröder et al., 2013). Moreover, the IEB data itself and the nature of the data that the IEB provides, are one important strength of this study. The register data is not subject to possible recall bias. This is a relevant advantage compared to most previous studies that used self-reported data. In addition, the availability of information on a daily basis regarding many variables can be seen as another strength of the study. As a result, income trajectories could be calculated more precisely, compared to many previous studies. Furthermore, in Germany, income is used to calculate the amount of social benefit accruing to each person and therefore represents highly valid information. A further major advantage of our study is represented in our long observation period of 24 years. Only a few studies have applied the life course approach to examine the complexity of the GPG. Our life course data contain various information about employment characteristics which are relevant for the GPG and of high data quality.

Our results showed, even after controlling for relevant factors, that the GPG still persisted. There exist some explanations of the GPG regarding different behaviors of men and women in wage negotiations, which further influence different income developments (Boll and Leppin, 2015). Also, structural disadvantages in the labor market can be a factor explaining the GPG. Individual behavior and labor market structures are not represented in our register data. We can only extract information that is relevant for social security contribution. Nonetheless, previous research of Blau and Kahn (2017) found a larger and more slowly decreasing GPG in the US at the top compared to other levels of the wage distribution. This ‘glass ceiling effect’ describes the reduced career opportunities of women compared to men due to frequent denial of access to leadership positions. Consequently, gender inequality can be found to be greater at the top of the wage distribution. Among European countries, previous studies have found this “glass ceiling effect” in Germany as well (Arulampalam et al., 2005; Boll and Leppin, 2015; Huffman et al., 2017). However, recent results of Boll et al. (2017) could not confirm the glass ceiling effect in West Germany, thus further research is needed.

## 5 Conclusion

The gender pay inequalities in the German labor market from a life course perspective exist. Our results demonstrated that human capital determinants continue to be important in explaining the GPG over time. Furthermore, factors of working disadvantages such as marginal work or unemployment are important when trying to explain the income differences of men and women. For further research the availability of more work data over the life course with matching individual data would help to understand the GPG even better.

## Data Availability

The datasets presented in this article are not readily available because the study data contain social security information. Due to legal regulations in Germany, it is not permitted to share data with social security information. Requests to access the datasets should be directed to lisa.toczek@uni-ulm.de.
