# Supplementary material for: The Gender Pay Gap: Income Inequality Over Life Course – A Multilevel Analysis
Source: Front Sociol. 2021 Dec 23;6:815376. doi: 10.3389/fsoc.2021.815376 (PMC8733696; doi:10.3389/fsoc.2021.815376)
Supplement: Supplementary file 1 [file DataSheet1.docx]

**Supplementary Data**

**Table S1** Definition of the characteristics of the variable education and vocational training.

|  | |  | Education | | | | | |
| --- | --- | --- | --- | --- | --- | --- | --- | --- |
|  |  |  | No qualification | Lower secondary school (8/9 years) | Secondary school (10 years) | Technical college (12/13 years) | Academic secondary school (12/13 years) | Other qualification |
| Vocational training | No qualification |  | 1 | 1 | 1 | 2 | 2 | 1 |
|  | Vocational school – company |  | 1 | 1 | 2 | 2 | 2 | 1 |
|  | Vocational school – college |  | 1 | 1 | 2 | 2 | 2 | 1 |
|  | Specialized secondary school |  | - | 2 | 2 | 2 | 2 | 2 |
|  | University of applied sciences |  | - | - | 3 | 3 | 3 | 3 |
|  | University |  | - | - | 3 | 3 | 3 | 3 |
|  | Other qualification |  | - | 1 | 2 | 2 | 3 | 1 |
